# Burden of COVID‐19 disease in Kurdistan province in west of Iran using disability‐adjusted life years

**DOI:** 10.1002/hsr2.1154

**Published:** 2023-03-22

**Authors:** Cyrus Alinia, Amjad M. Bolbanabad, Ghobad Moradi, Azad Shokri, Ebrahim Ghaderi, Jalil Adabi, Satar Rezaei, Bakhtiar Piroozi

**Affiliations:** ^1^ Healthcare Management & Economics Department, School of Public Health Urmia University of Medical Sciences Urmia Iran; ^2^ Social Determinants of Health Research Center, Research Institute for Health Development Kurdistan University of Medical Sciences Sanandaj Iran; ^3^ School of Public Health Kermanshah University of Medical Sciences Kermanshah Iran

**Keywords:** burden of disease, COVID‐19, disability‐adjusted life years, Iran, years of life lost

## Abstract

**Background and Aims:**

During the coronavirus disease 2019 (COVID‐19) pandemic, about seven million people were infected with the disease, of which more than 133,000 died. Health policymakers need to know the extent and magnitude of the disease burden to decide on how much to allocate resources for disease control. The results of this investigation could be helpful in this field.

**Methods:**

We used the secondary data released by the Kurdistan University of Medical Sciences between February 2020 to October 2021 to estimate the age‐sex standardized disability‐adjusted life years (DALY) by the sum of the years of life lived with disability (YLD) and the years of life lost (YLL). We also applied the local and specific values of the disease utility in the calculations.

**Results:**

The total DALY was estimated at 23316.5 and 1385.5 per 100,000 populations The YLD and YLL constituted 1% and 99% of the total DALY, respectively. The DALY per 100,000 populations was highest in the men and people aged more than 65 years, but the prevalence was the highest in people under the age of 40.

**Conclusions:**

Compared to the findings of the “burden of disease study 2019,” the burden of COVID‐19 in Iran is ranked first and eighth among communicable and noncommunicable diseases, respectively. Although the disease affects all groups, the elderly suffer the most from it. Given the very high YLL of COVID‐19, the best strategy to reduce the burden of COVID‐19 in subsequent waves should be to focus on preventing infection in the elderly population and reducing mortality.

## 1 INTRODUCTION

The coronavirus disease 2019 (COVID‐19) pandemic has spread in the world and has become a serious challenge for governments and health systems, so that countries have experienced several waves of this disease so far.^1^, ^2^ As of February 14, 2022, the new coronavirus has infected more than 413 million people and killed 5.8 million people worldwide. The coronavirus pandemic was the second leading cause of death in Oceania, the third in North America and the 19th leading cause of death in Asia.^3^, ^4^ Iran is also one of the countries most affected by the virus, so that to date, the virus has infected more than 6.8 million people and killed more than 133,000 people.^3^, ^4^

In addition to the epidemiological burden, the COVID‐19 pandemic has imposed a significant economic burden on countries around the world. Thus, in 2020 alone, it led to the loss of 6.65% of GDP in the world, and if the pandemic continues, this amount will reach 48% in 2020–2030.^5^ Low‐ and middle‐income countries, due to lack of adequate financial support, are unable to support the vulnerable and this has led to a poorer society and an increase in the death rate from COVID‐19.^6^, ^7^ However, little is known about the socioeconomic impact of COVID‐19 in low‐ and middle‐income countries.^8^, ^9^ To estimate these effects, DALYs can be a useful tool.^10^, ^11^ This index shows the rates of premature death and the inability caused by the disease to quantify the burden of the disease.^12^ DALYs, which is one of the adjusted life expectancy indicators, is an indicator for COVID‐19 to judge the success of preventive and curative interventions in health systems, allowing policymakers to make standard comparisons between different health systems. In addition, this index determines the extent and rank of the epidemiological burden of the disease in comparison with other common diseases within any health system.^13^, ^14^ Utilizing the results of burden of disease studies will lead to optimal and cost‐effective decisions that will benefit society the most.^15^

Researchers have calculated the burden of the epidemiological disease of COVID‐19 for some countries. These calculations are different in terms of the value of the disease utility (specific/nonspecific), the extent of the geographical area (international/national/regional/provincial), and the period of the study (the first or second wave of the disease/the entire period of the disease). These differences have made the disease burden of COVID‐19 not only dependent on the prevalence of the disease but also affected by the study methodology. So the results cannot be directly compared with each other.^16^, ^17^, ^18^, ^19^, ^20^, ^21^, ^22^, ^23^, ^24^, ^25^, ^26^, ^27^ In addition, studies conducted in the United States^28^ and Japan^29^ have investigated the effect of the outbreak of the COVID‐19 pandemic on changes in out‐of‐hospital cardiac arrest and other vaccine‐preventable diseases DALYs, respectively.

The aim of this study was to describe and estimate the epidemiological burden of COVID‐19 in Kurdistan province in west of Iran. What sets this study apart from other studies is the use of the COVID‐19‐specific disability weight.^30^ For the first time, this study has calculated the burden of COVID‐19 disease in Iran using local and specific disability weight values of this disease, which informs health policy makers about the extent of the disease in comparison with other communicable and noncommunicable diseases. The findings of this study can determine the position of the COVID‐19 disease in the country and provide enough justification for allocating sufficient funds and resources to control this vaccine‐preventable disease burden.

## 2 MATERIALS AND METHODS

### 2.1 Study design and participants

This retrospective descriptive epidemiological study was conducted at Kurdistan province, in the west of Iran, covered all laboratory‐confirmed cases during February 2020 to October 2021. This border province has an area of 29,137 square kilometers and a population of more than 1.6 million people. About a third of this population lives in rural areas.^31^

In accordance with the World Health Organization (WHO) recommendations, we defined people with COVID‐19 disease as having a positive reverse transcriptase polymerase chain reaction (RT‐PCR) and included data from all laboratory‐confirmed cases during the study period. According to the recommendations of the WHO, qualitative real‐time RT‐PCR nasopharyngeal swab which detects the presence of specific segments of the SARS‐CoV‐2 genome is the gold standard method for diagnosing COVID‐19 disease. This diagnostic approach has high specificity but moderate sensitivity of 50%–62%.^32^ Therefore, the presence of substantial false negatives confirms that the confirmed cases are significantly less than the actual cases with COVID‐19.

According to the national protocols, RT‐PCR test is taken from the suspected cases. These cases are people who have one or more signs and symptoms of the disease including fever, cough, general weakness/fatigue, headache, myalgia, sore throat, coryza, dyspnea, anorexia/nausea/vomiting, diarrhea, and altered mental status or those have been in close contact with the infected people for the past 14 days, including all people who have lived with them.

### 2.2 Data collection

We gathered data on COVID‐19's outpatients from the Deputy of Health and the inpatients from the Health Information System of the Deputy of Treatment of Kurdistan University of Medical Sciences. This data is collected daily from testing centers and designated hospitals for the management of patients with COVID‐19 and aggregated in the mentioned deputies. We considered 14 days as the average quarantine time at home for the outpatients and discharged patients according to the national protocol.

### 2.3 Modeling

Using DISMOD 11 software,^33^ we estimated the DALYs value and rates per 100,000 by summing the years of life lived with disability (YLDs) and the years of life lost (YLLs) due to premature death^15^ as follows:

DALY = YLDs + YLLs.

Where YLDs equals the sum of years spent with disability due to COVID‐19 disease. Disability is defined as reduced HRQoL attributed to the diseases.^30^ To calculate YLDs, the annual equivalent of number of days spent with the disease is multiplied by the value of the disease disutility (=1‐utility). The disease duration was equal to the time interval between the onset of symptoms or signs until complete recovery or before death.^15^ Finally, the sum of YLDs obtained for each infected person showed the number of YLDs in the community.

Also, YLLs metrics were calculated by taking the product of the number of deaths from age‐sex‐specific estimates of mortality by COVID‐19 disease. Estimated Iranian life expectancy was obtained from WHO statistics.^34^ The YLLs were summed across age categories to determine total YLLs. Due to the fact that the COVID‐19 disease has a different effect on young, middle‐aged, and the elderly, we have presented the results in terms of three age groups of under 40, 40–65, and over 65 years.

According to the findings of our previous study, the health status of patients with the COVID‐19 is divided into four groups based on the severity of the disease, which determines the level of health‐related quality of life: Quarantine at home, general wards hospitalization, ICU hospitalization with no intubation, and ICU hospitalization with intubation. Using the time trade‐off approach, the average utility values of these patients were estimated as 0.896, 0.847, 0.766, and 0.629, respectively.^30^

### 2.4 Ethical considerations

The ethics committee of Kurdistan University of Medical Sciences approved the protocol of the study (No. IR.MUK.REC.1399.077). The researchers explained research goals and protocol to the participants before their inclusion in the study, and written informed consent was obtained from all eligible participants who were willing to participate in the study.

## 3 RESULTS

The total number of confirmed cases during the study period was 53550 people, including 33,616 outpatients and 19,934 hospitalized cases. About 8% of the hospitalized cases, 1592 cases (862 males and 730 females), were admitted to the intensive care unit, of which 21.5%, 342 cases (183 males and 159 females), were intubated. In addition, 2030 people (1143 men and 887 women) that is equivalent to 3.79% of the total number of patients and 10.18% of hospitalized cases died due to COVID‐19. The mean length of hospital stay for COVID‐19 patients admitted to the general ward, ICU ward, and hospitalized with intubation were 5.34, 8.87, and 6.87 days, respectively. The mean (standard deviation) age of inpatients and outpatients was 54.3 (19.16) and 38.2 (18.2) years, respectively. Also, the highest rate of infection (40.2%) and the highest rate of mortality (65.5%) were observed in <40 and over 65 age groups, respectively. Fatality rate was 0.47% for people under 40, 3.02% for people aged 40–65 and 10.91% for people over 65.

In total, COVID‐19 imposed 23316.5 DALYs on the people of Kurdistan Province during the study period, of which about 99% was due to YLL and only the remaining 1% was due to YLD. About 54.7% of this DALY belonged to men and 45.3% belonged to women. Also, most of the epidemiological burden) 45%) was related to patients over 65 years old, 42.8% was related to 40–65 age group and 12.2% was related to <40 age group.

According to the findings, COVID‐19 resulted in an average loss of 0.435 years per person. Findings indicated that COVID‐19 imposed 1385.3 DALYs per 100,000 populations, which was 11299.3, 2354.4, and 243.5 DALYs for patients older than 65 years, between 40 and 65 years and less than 40 years, respectively (Table 1).

**Table 1:** Years of life lost (YLL), years lost due to disability (YLD), and disability‐adjusted life years (DALY) attributable to COVID‐19 by gender, age groups, and patient's condition in Kurdistan province, Iran.

Variable	Epidemiological metrics						
	Confirmed cases	Deaths	YLD	YLL	DALY	DALY for each COVID‐19 case	DALY per 100,000 population
Gender							
Male	27,085	1143	128.4	12622.5	12750.9	0.470	1494.1
Female	26,465	887	127.8	10437.8	10565.6	0.399	1273.4
Age group							
<40 years	21,557	102	96.6	2748.3	2844.9	0.131	243.5
40–65 years	19,798	598	99.3	9887.1	9986.4	0.504	2365.4
>65 years	12,195	1330	60.3	10424.9	10485.2	0.859	11299.3
Patient's condition							
Outpatient	33,616	0	134.2	0	134.2	0.004	‐
Inpatient	19,934	2030	122.0	23060.3	23182.3	1.163	‐
Total	53,550	2030	256.2	23060.3	23316.5	0.435	1385.3

Due to the uncertainty about the variables of the disease prevalence rate, the number of quarantine days of patients with a positive COVID‐19 test in all disease conditions and having the utility value related to different degrees of the disease, one‐way sensitivity analysis was performed on these parameters. According to experts, between 25% and 100% of positive COVID‐19 cases in Iran are under‐estimated. Therefore, although the total number of DALYs of the disease imposed on people in Kurdistan province was equal to 23316.5 in the basic model until October 2021, taking into account the mentioned under‐estimation rate, this amount reaches a minimum of 29145.6 and a maximum of 46633 DALYs.

Also, if we consider the interval rate of the number of quarantine days at home between 10 and 18 days, the total amount of DALYs imposed on the population of the province will vary in the range of 23375.1–23257.7 years. In addition, if we consider the 95% confidence interval introduced for utility values of different degrees of disease in the study of Alinia et al.^30^ in the calculations of this study, then, COVID‐19 has imposed between 23404 and 23228.7 DALYs on the population of the province (Table 2).

**Table 2:** Results of one‐way sensitivity analysis of epidemiological burden of COVID‐19 disease in Kurdistan province, Iran.

Parameters		Lower limit	Upper limit	Disability‐adjusted life years (DALY) range	
				Minimum	Maximum
Prevalence underestimation (percent)		25	100	29145.6	46633.0
Quarantine duration (days)		10	18	23257.7	23375.1
Utility value	*Quarantine at home*	0.857	0.935	23228.7	23404.0
	*General wards hospitalization*	0.827	0.867		
	*ICU hospitalization with no intubation*	0.648	0.884		
	*ICU hospitalization with intubation*	0.374	0.884		

## 4 DISCUSSION

The COVID‐19 pandemic has significantly reduced the well‐being of communities by imposing significant epidemiological and economic burdens on countries, and today the disease is considered to be one of the leading causes of death.^35^, ^36^, ^37^ However, the epidemiological effects of the disease are not limited to death; the morbidity has also placed many limitations on the patient's health‐related quality of life and those around them.^38^, ^39^, ^40^, ^41^, ^42^ In our previous study,^30^ we categorized the disease according to severity and showed it can destroy an average of 13.7% of the health‐related quality of life. For the first time, using this specific utility value, we have calculated and presented the burden of COVID‐19 disease for Iran.

According to the findings, from February 2020 to October 2021, a total of 53550 people (50.6% males) were infected with COVID‐19 based on the results of PCR test and of these 2030 people (56.3% males) died in Kurdistan province. 37.2% of total patients were hospitalized. The findings indicated that the total DALYs due to COVID‐19 was about 23,316 and the amount of adjusted DALYs per 100,000 populations was equal to 1385 years for a period of 20 months. Assuming that the incidence rates of the disease were evenly distributed throughout the study period, its DALYs index for a one‐year was 831 per 100,000 population, which is equivalent to 3.5% of the total DALYs of all causes (23,522.2 DALYs per 100,000 population) for Iranians based on the Global Burden of Disease (GBD) study in 2019.^43^ These findings are clearly a vibrant answer for the shocks that COVID‐19 has inflicted on communities. Various waves of the disease led to the filling of the capacity of hospital beds and imposed a lot of work pressure on doctors, nurses and other healthcare providers.^44^, ^45^

Tables 3 and 4 are presented to better understand the epidemiological burden of COVID‐19 and the resulting pressure on the Iranian health system. Table 3 shows the adjusted burden of disease per 100,000 population for a number of noncommunicable diseases with the highest DALYs at national level based on the GBD study, 2019.^43^ As can be seen in this Table, the burden of COVID‐19 disease is in the eighth place compared to other diseases (Table 3).

**Table 3:** Burden of COVID‐19 disease compared with the noncommunicable diseases with the highest burden in Iran.

Diseases	Disability‐adjusted life years (DALY) per 100,000 population	COVID‐19 ratio to others
COVID‐19	831.0	1
Ischemic heart disease	2402.7	0.35
Road injuries	1320.0	0.63
Stroke	1049.6	0.79
Neonatal disorders	1009.9	0.82
Low back pain	993.8	0.84
Depressive disorders	965.0	0.86
Diabetes	902.2	0.92
Congenital defects	613.3	1.33

*Source*: Institute for Health Metrics and Evaluation, Burden of disease study, 2019.^43^

**Table 4:** Burden of COVID‐19 disease compared with other communicable diseases with the highest burden in Iran.

Disease	Disability‐adjusted life years (DALY) per 100,000 population	COVID‐19 ratio to others
COVID‐19	831.0	1
Communicable, maternal, neonatal, and nutritional diseases	2106.8	0.39
Respiratory infections and tuberculosis	445.0	1.87
HIV/AIDS	76.0	8.67
Tuberculosis	32.2	25.8
Acute hepatitis	22.1	37.60
Acute hepatitis B	16.5	50.37

*Source*: Institute for Health Metrics and Evaluation, Burden of disease study, 2019.^43^

Table 4 presents the adjusted burden of disease per 100,000 population for a number of communicable diseases with the highest DALYs at national level based on the GBD study in 2019.^43^ As can be seen in this Table, burden of COVID‐19 is in the first place compared to the burden of other diseases and it has about twice the burden of respiratory infections and tuberculosis. Also, the epidemiological burden of this disease alone is equal to 39.4% of the total infectious diseases, mother and infant, and nutrition‐related diseases (2106.8 DALYs per 100,000 people) in the country (Table 4).

The range of DALYs per 100,000 populations is wide in different studies and is probably influenced by the timing of the study, government interventions, policy and public health measures, and the combined effect of demographic, epidemiological, and cultural variables of countries.^13^, ^14^, ^45^ For instance, in a study by Guru et al. in the Indian state of Maharashtra, the DALYs generated by COVID‐19 was 600 per 100,000 people by December 2020.^25^ Another study conducted in 16 European countries between January 2020 and November 2020 found the highest DALYs per 100,000 people in Italy (650), the Czech Republic (534) and Sweden (529).^13^ In another study in 30 countries with a high prevalence of COVID‐19, the highest DALYs per 100,000 people was observed in Belgium (1593.7).^14^

The findings of our study showed that the share of YLL from burden of COVID‐19 disease was about 99%; indicating a significant mortality and a high share of burden of disease. This finding is consistent with the findings of other studies. For example, in a study by Min‐Woo Jo et al. in South Korea, the YLL's share of total COVID‐19‐induced DALYs was 89.7%,^46^ and it were more than 98% in another studies in 16 European countries,^13^ in India,^22^ in Colombia,^18^ in Germany,^21^ and in Netherland.^19^

Similar to other related investigations, the highest crude and population‐adjusted DALYs were observed in men and those group over 65 age. The reason is that the higher mortality rate was concentrated among men (56.3%) and elderly people (65.5%). The elderly have become more vulnerable to COVID‐19 due to their increased risk of underlying diseases such as diabetes, high blood pressure, and chronic obstructive pulmonary disease.^47^, ^48^, ^49^ This finding of our study is consistent with the findings of other studies.^50^, ^51^ In a study of 30 countries with a high prevalence of COVID‐19 disease, the highest levels of YLL were concentrated in men and people over 60 years of age.^14^ In our study, about 99.4% of DALYs was attributed to hospitalized patients due to the high share of YLL in DALYs because of premature death and the low share of YLD due to the short duration of the disease, especially in outpatients. It is expected that in the study period, the number of DALYs due to COVID‐19 in Kurdistan province be more than the amount calculated based on the recorded cases and official reports because of under‐estimation and undiscovered cases of the disease. Therefore, in this study, sensitivity analyzes were performed to evaluate the effect of the assumptions on the range of DALYs estimates. The most significant change in the total amount of DALYs was observed due to the change in the prevalence of the disease, which indicates a very high share of YLL from burden of COVID‐19 disease.

When the confirmed cases of disease increases by 25%–100%, the DALYs value also increases by approximately the same amount (in the range of 29,245–46,633). Sensitivity of DALYs value to disease duration and disease disability weight was low. For example, applying the highest disability weight and the highest quarantine duration for COVID‐19 resulted in only a 0.37% and 0.25% increase in DALYs, respectively. Sensitivity analysis of a study conducted in South Korea on COVID‐19 patients showed that with an increase of 10%, 20%, and 30% in the number of deaths, the total DALYs increased by 9%, 17.9%, and 26.9%, respectively.^46^

### 4.1 Strengths and limitations

It is important to bear in mind the possible bias in the findings. The study data was only related to cases with positive PCR test and did not covers those with false‐negative tests and infected people who did not give any diagnostic tests. So, obtained DALY value may be underestimated. To overcome this severe uncertainty, we performed a one‐way sensitivity analysis over the key factors including COVID‐19 disease's prevalence rate, quarantine time, and the utility values. The analysis determined the range of total amount of DALYs attributed to COVID‐19 disease to be 23228.7–46633.0 in Kurdistan province. This finding was mainly affected by the prevalence rate parameter.

Nonetheless, this study applied the specific, domestic, and health state disability weights in the calculations for the first time and estimated a more validated epidemiological burden compared with similar investigations. Other related studies used a fixed disutility value of other acute lower respiratory tract infections as a proxy equal to 0.133. Even though this value is very close to the mean of specific disability weight that we used in this study, the weights for the severe conditions like ICU hospitalization (0.234) and with intubation (0.371) are significantly higher and impose more burdens on the patients. In addition to considering the disease grading and specific disability values, we also applied an age‐sex‐specific life expectancy to standardize the COVID‐19 DALY. Such considerations were not seen in other similar studies that could result in underestimation of the disease burden. It is suggested to repeat this study at the provincial and national levels for different outbreak waves to monitor the changes in the burden of the disease of COVID‐19 in the future.

### 4.2 Policy implications

Iran has experienced a relatively more prevalence and mortality rate related to COVID‐19 disease compared to the global average^52^ and its impact is reflected in the disease's burden. This problem has two main sources; insufficient observance of preventive behaviors and insufficient effectiveness of therapeutic interventions.^52^, ^53^, ^54^ Adult vaccination strategy began late in Iran and COVID‐19 waves led to higher hospitalization and deaths in most countries. Since the majority of the calculated DALY is associated with the YLL, the value of preventive behaviors such as vaccination and boosters, wearing a face mask, observing the social distance, and other effective actions are more highlighted in the decrease of disease burden. So far, only about 65 percent of the population has received at least two doses of the vaccine,^4^ indicating the fragility of the herd immunity against subsequent COVID‐19 waves and variants. Also, it seems that we have a considerable underestimation in daily incidence reporting because of taking a low number of PCR tests and its high false‐negative rates. So, the health decision‐makers should expand the vaccination program coverage as well as case‐finding policies, and update the therapeutic interventions to achieve the best health outcomes and control the burden of disease.

## 5 CONCLUSION

To know the extent, burden, and rank of COVID‐19 among all diseases, we conducted this study using the comprehensive data of patients registered during the entire pandemic period and also using specific and local values of the disease's utility and we were able to provide a valid understanding of the disease epidemiological burden. The burden of COVID‐19‐related DALY was 831 per 100,000 populations in 12 months in Kurdistan province. The YLL and the YLD constituted 99% and 1% of the total DALY, respectively. This burden of disease is considerably high compared with the global average and it ranks first among communicable, maternal, neonatal, and nutritional diseases and eighth among all of the diseases with high burden in Iran based on the findings of the GBD study. This place will be upgraded to the second rank if we accept the results of upper bound of sensitivity analysis that overcame the underestimation bias in disease incidence. The obtained DALY is almost concentrated among the elderly people and is because of years of life lost due to premature deaths. The most effective strategy to control the burden of COVID‐19 disease is to developing vaccination coverage as soon as possible, detecting patients promptly, and using more effective treatments.

## AUTHOR CONTRIBUTIONS

**Cyrus Alinia**: Conceptualization; formal analysis; methodology; visualization; writing—original draft. **Amjad M. Bolbanabad**: Conceptualization; formal analysis; funding acquisition; investigation; supervision; visualization; writing—original draft. **Ghobad Moradi**: Conceptualization; formal analysis; methodology; visualization; writing—original draft. **Azad Shokri**: Data curation; formal analysis; methodology; project administration; writing—original draft. **Ebrahim Ghaderi**: Conceptualization; investigation; methodology; writing—original draft. **Jalil Adabi**: Data curation; project administration; resources; validation; visualization; writing—original draft. **Satar Rezaei**: Formal analysis; methodology; visualization; writing—original draft. **Bakhtiar Piroozi**: Conceptualization; formal analysis; investigation; methodology; project administration; visualization; writing—original draft.

## CONFLICT OF INTEREST STATEMENT

The authors declare no conflict of interest.

## ETHICS STATEMENT

This study was approved by ethics committee of Kurdistan University of Medical Sciences with the code IR.MUK.REC.1399.077. Participation in the study was voluntary and written consent was obtained from the participants.

## TRANSPARENCY STATEMENT

The lead author Bakhtiar Piroozi affirms that this manuscript is an honest, accurate, and transparent account of the study being reported; that no important aspects of the study have been omitted; and that any discrepancies from the study as planned (and, if relevant, registered) have been explained.

## Data Availability

The data that support the findings of this study are available from the corresponding author (Bakhtiar Piroozi) upon reasonable request.

## References

[1] Armocida B , Formenti B , Ussai S , Palestra F , Missoni E . The Italian health system and the COVID‐19 challenge. Lancet Public Health. 2020;5(5):e253. 32220653 10.1016/S2468-2667(20)30074-8 PMC7104094

[2] Gualano MR , Lo Moro G , Voglino G , Bert F , Siliquini R . Monitoring the impact of COVID‐19 pandemic on mental health: a public health challenge? reflection on Italian data. Soc Psychiatry Psychiatr Epidemiol. 2021;56(1):165‐167. 33034669 10.1007/s00127-020-01971-0 PMC7545814

[3] Worldometer . *COVID‐19 Coronavirus Pandemic*; 2022. https://wwwworldometersinfo/coronavirus/

[4] World Health Organization . *WHO Coronavirus (COVID‐19) Dashboard*. World Health Organization. https://covid19.who.int/

[5] Jackson JK . Global economic effects of COVID‐19. Congression Res Service. 2021. https://sgp.fas.org/crs/row/R46270.pdf

[6] Bong C‐L , Brasher C , Chikumba E , McDougall R , Mellin‐Olsen J , Enright A . The COVID‐19 pandemic: effects on low‐and middle‐income countries. Anesth Analg. 2020;1:86‐92. 10.1213/ANE.0000000000004846 PMC7173081 32243287

[7] Walker PGT , Whittaker C , Watson OJ , et al. The impact of COVID‐19 and strategies for mitigation and suppression in low‐and middle‐income countries. Science. 2020;369(6502):413‐422. 32532802 10.1126/science.abc0035 PMC7292504

[8] Cuschieri S , Calleja N , Devleesschauwer B , Wyper GMA . Estimating the direct Covid‐19 disability‐adjusted life years impact on the Malta population for the first full year. BMC Public Health. 2021;21(1):1827. 34627228 10.1186/s12889-021-11893-4 PMC8501913

[9] Singh BB , Devleesschauwer B , Khatkar MS , et al. Disability‐adjusted life years (DALYs) due to the direct health impact of COVID‐19 in India, 2020. Sci Rep. 2022;12(1):2454. 35165362 10.1038/s41598-022-06505-z PMC8844028

[10] Salinas‐Escudero G , Toledano‐Toledano F , García‐Peña C , Parra Rodríguez L , Granados‐García V , Carrillo Vega MF . Disability‐adjusted life years for the COVID‐19 pandemic in the Mexican population. Front Public Health. 2021;9:1077. 10.3389/fpubh.2021.686700 PMC8415975 34485216

[11] Wyper GMA , Assunção RMA , Colzani E , et al. Burden of disease methods: a guide to calculate COVID‐19 disability‐adjusted life years. Int J Public Health. 2021;66:1‐5. 10.3389/ijph.2021.619011 PMC8565264 34744580

[12] Fan C‐Y , Fann JC‐Y , Yang M‐C , et al. Estimating global burden of COVID‐19 with disability‐adjusted life years and value of statistical life metrics. J Formos Med Assoc. 2021;120:S106‐S117. 34119392 10.1016/j.jfma.2021.05.019 PMC8165085

[13] Gianino MM , Savatteri A , Politano G , Nurchis MC , Pascucci D , Damiani G . Burden of COVID‐19: disability‐adjusted life years (DALYs) across 16 European countries. Eur Rev Med Pharmacol Sci. 2021;25:5529‐5541. 34533803 10.26355/eurrev_202109_26665

[14] Oh I‐H , Ock M , Jang SY , et al. Years of life lost attributable to COVID‐19 in high‐incidence countries. J Korean Med Sci. 2020;35(32):1‐10. 10.3346/jkms.2020.35.e300 PMC7431288 32808515

[15] Murray CJL , Vos T , Lozano R , et al. Disability‐adjusted life years (DALYs) for 291 diseases and injuries in 21 regions, 1990–2010: a systematic analysis for the global burden of disease study 2010. Lancet. 2012;380(9859):2197‐2223. 23245608 10.1016/S0140-6736(12)61689-4

[16] Asdaq SMB , Rabbani SI , Alshammari MK , et al. Burden of COVID‐19: a preliminary analysis in the population of Saudi Arabia. PeerJ. 2022;10:e13219. 35415012 10.7717/peerj.13219 PMC8995037

[17] He M , Li X , Tan Q , et al. Disease burden from COVID‐19 symptoms among inpatients at the temporary military hospitals in Wuhan: a retrospective multicentre cross‐sectional study. BMJ Open. 2021;11(5):e048822. 10.1136/bmjopen-2021-048822 PMC8130755 34006559

[18] Hidalgo‐Troya A , Rodríguez JM , Rocha‐Buelvas A , Urrego‐Ricaurte D . Burden of disease of COVID‐19 in the department of Nariño, Colombia, 2020‐2021. Revista Peruana de Medicina Experimental y Salud Publica. 2022;39(3):281‐291. 36478161 10.17843/rpmesp.2022.393.10947 PMC11397680

[19] McDonald SA , Lagerweij GR , de Boer P , et al. The estimated disease burden of acute COVID‐19 in the Netherlands in 2020, in disability‐adjusted life‐years. Eur J Epidemiol. 2022;37(10):1035‐1047. 35951278 10.1007/s10654-022-00895-0 PMC9366822

[20] Nurchis MC , Pascucci D , Sapienza M , et al. Impact of the burden of COVID‐19 in Italy: results of disability‐adjusted life years (DALYs) and productivity loss. Int J Environ Res Public Health. 2020;17(12):4233. 32545827 10.3390/ijerph17124233 PMC7345321

[21] Rommel A , Lippe EV , Plass D , et al. The COVID‐19 disease burden in Germany in 2020—Years of life lost to death and disease over the course of the pandemic. Dtsch Arztebl Int. 2021;118(9):145‐151. 33958032 10.3238/arztebl.m2021.0147 PMC8212397

[22] Shindhe S , Bhat S , Munoli S . Burden of COVID‐19: DALY and productivity loss for Karnataka, India. Indian J Public Health. 2022;66(3):239‐244. 36149098 10.4103/ijph.ijph_959_21

[23] Smith MP . Estimating total morbidity burden of COVID‐19: relative importance of death and disability. JCE. 2022;142:54‐59. 34715312 10.1016/j.jclinepi.2021.10.018 PMC8547965

[24] Taheri Soodejani M , Abedi Gheshlaghi L , Bahrevar V , Hosseini S , Lotfi MH . Burden of severe COVID‐19 in center of Iran: results of disability‐adjusted life years (DALYs). Int J Mol Epidemiol Genet. 2021;12(6):120‐125. 35126835 PMC8784907

[25] Vasishtha G , Mohanty SK , Mishra US , Dubey M , Sahoo U . Impact of COVID‐19 infection on life expectancy, premature mortality, and DALY in Maharashtra, India. BMC Infect Dis. 2021;21(1):343. 33845774 10.1186/s12879-021-06026-6 PMC8040360

[26] Wyper GMA , Assunção R , Cuschieri S , et al. Population vulnerability to COVID‐19 in Europe: a burden of disease analysis. Arch Public Health. 2020;78:47. 32501409 10.1186/s13690-020-00433-y PMC7256342

[27] Zhao J , Jin H , Li X , et al. Disease burden attributable to the first wave of COVID‐19 in China and the effect of timing on the cost‐effectiveness of movement restriction policies. Value Health. 2021;24(5):615‐624. 33933229 10.1016/j.jval.2020.12.009 PMC7897405

[28] Coute RA , Nathanson BH , Kurz MC , Mader TJ . Estimating the impact of the COVID‐19 pandemic on out‐of‐hospital cardiac arrest burden of disease in the United States. J Am Coll Emer Phys Open. 2022;3(5):e12811. 10.1002/emp2.12811 PMC9445427 36090004

[29] Kitano T . The estimated burden of 15 vaccine‐preventable diseases from 2008 to 2020 in Japan: a transition by the COVID‐19 pandemic. J Infect Chemother. 2021;27(10):1482‐1488. 34244054 10.1016/j.jiac.2021.06.021 PMC10130821

[30] Alinia C , Yaghmaei S , Abdullah FZ , et al. The health‐related quality of life in Iranian patients with COVID‐19. BMC Infect Dis. 2021;21(1):1‐8. 34016041 10.1186/s12879-021-06170-z PMC8135385

[31] Statistical Centre of Iran . *Population and Housing Censuses*. Statistical Centre of Iran; 2011. https://www.amar.org.ir/english/Population-and-Housing-Censuses

[32] Vinh DB , Zhao X , Kiong KL , et al. Overview of COVID‐19 testing and implications for otolaryngologists. Head Neck. 2020;42(7):1629‐1633. 32342570 10.1002/hed.26213 PMC7267427

[33] Barendregt JJ , Van Oortmarssen GJ , Vos T , Murray CJ . A generic model for the assessment of disease epidemiology: the computational basis of DisMod II. Popul Health Metr. 2003;1(1):4. 12773212 10.1186/1478-7954-1-4 PMC156029

[34] World Health Organization . *Life tables by country Islamic Republic of Iran*. World Health Organization; 2013. https://apps.who.int/gho/data/?theme=main&vid=60760

[35] Di Fusco M , Shea KM , Lin J , et al. Health outcomes and economic burden of hospitalized COVID‐19 patients in the United States. J Med Econ. 2021;24(1):308‐317. 33555956 10.1080/13696998.2021.1886109

[36] Jin H , Wang H , Li X , et al. Economic burden of COVID‐19, China, January–March, 2020: a cost‐of‐illness study. Bull World Health Organ. 2021;99(2):112‐124. 33551505 10.2471/BLT.20.267112 PMC7856360

[37] Darab MG , Keshavarz K , Sadeghi E , Shahmohamadi J , Kavosi Z . The economic burden of coronavirus disease 2019 (COVID‐19): evidence from Iran. BMC Health Serv Res. 2021;21(1):1‐7. 33573650 10.1186/s12913-021-06126-8 PMC7877330

[38] Ahmad S , Shoaib A , Ali MS , et al. Epidemiology, risk, myths, pharmacotherapeutic management and socio economic burden due to novel COVID‐19: a recent update. Res J Pharm Technol. 2020;13(9):4435‐4442.

[39] Oberndorfer M , Dorner TE , Brunnmayr M , Berger K , Dugandzic B , Bach M . Health‐related and socio‐economic burden of the COVID‐19 pandemic in Vienna. Health Soc Care Commun. 2021;4:1550‐1561. 10.1111/hsc.13485 PMC8444637 34219320

[40] Ferreira LN , Pereira LN , da Fé Brás M , Ilchuk K . Quality of life under the COVID‐19 quarantine. Qual Life Res. 2021;30(5):1389‐1405. 33389523 10.1007/s11136-020-02724-x PMC7778495

[41] Chen K‐Y , Li T , Gong F‐H , Zhang J‐S , Li X‐K . Predictors of health‐related quality of life and influencing factors for COVID‐19 patients, a follow‐up at one month. Front Psychiatry. 2020;11:668. 32733299 10.3389/fpsyt.2020.00668 PMC7360857

[42] Geirdal AØ , Ruffolo M , Leung J , et al. Mental health, quality of life, wellbeing, loneliness and use of social media in a time of social distancing during the COVID‐19 outbreak. A cross‐country comparative study. J Ment Health. 2021;30(2):148‐155. 33689546 10.1080/09638237.2021.1875413

[43] IHME . *Iran Islamic Republic of Institute for Health Metrics and Evaluation*. 2019. https://www.healthdata.org/iran

[44] Kalateh Sadati A , Zarei L , Shahabi S , et al. Nursing experiences of COVID‐19 outbreak in Iran: a qualitative study. Nurs Open. 2021;8(1):72‐79. 32904939 10.1002/nop2.604 PMC7461197

[45] Shoja E , Aghamohammadi V , Bazyar H , et al. Covid‐19 effects on the workload of Iranian healthcare workers. BMC Public Health. 2020;20(1):1636. 33138798 10.1186/s12889-020-09743-w PMC7605333

[46] Jo M‐W , Go D‐S , Kim R , et al. The burden of disease due to COVID‐19 in Korea using disability‐adjusted life years. J Korean Med Sci. 2020;35(21):1‐10. 10.3346/jkms.2020.35.e199 PMC7261698 32476305

[47] Azarpazhooh MR , Morovatdar N , Avan A , et al. COVID‐19 pandemic and burden of non‐communicable diseases: an ecological study on data of 185 countries. J Stroke Cereb Dis. 2020;29(9):105089. 10.1016/j.jstrokecerebrovasdis.2020.105089 PMC7315949 32807484

[48] Palmer K , Monaco A , Kivipelto M , et al. The potential long‐term impact of the COVID‐19 outbreak on patients with non‐communicable diseases in Europe: consequences for healthy ageing. Aging Clin Exp Res. 2020;32(7):1189‐1194. 32458356 10.1007/s40520-020-01601-4 PMC7248450

[49] Mistry SK , Ali ARMM , Yadav UN , et al. Older adults with non‐communicable chronic conditions and their health care access amid COVID‐19 pandemic in Bangladesh: findings from a cross‐sectional study. PLoS One. 2021;16(7):e0255534. 34324556 10.1371/journal.pone.0255534 PMC8320993

[50] Hashan MR , Smoll N , King C , et al. Epidemiology and clinical features of COVID‐19 outbreaks in aged care facilities: a systematic review and meta‐analysis. EClinicalMedicine. 2021;33:100771. 33681730 10.1016/j.eclinm.2021.100771 PMC7917447

[51] Holt NR , Neumann JT , McNeil JJ , Cheng AC . Implications of COVID‐19 for an ageing population. Med J Aust. 2020;213(8):342‐344. 32946607 10.5694/mja2.50785 PMC7537179

[52] Chaibakhsh S , Pourhoseingholi A , Vahedi M . Global incidence and mortality rate of COVID‐19; special focus on Iran, Italy and China. Arch Iran Med. 2020;23(7):455‐461. 32657596 10.34172/aim.2020.42

[53] Negahdaripour M . The rise and fall in therapeutic candidates for COVID‐19. Iran J Med Sci. 2020;45(4):231‐232. 32801412 10.30476/ijms.2020.46689 PMC7395951

[54] Koushki M , Abedipour F , Basiri N . Evaluation of the effectiveness of COVID‐19 treatments performed in Iran in comparison with other countries: a review. Acad J Health Sci Med Balear. 2021;36(4):65‐73.

